# Clusterin confers gmcitabine resistance in pancreatic cancer

**DOI:** 10.1186/1477-7819-9-59

**Published:** 2011-05-24

**Authors:** Qingfeng Chen, Zhengkun Wang, Kejun Zhang, Xiaoyi Liu, Weihong Cao, Lei Zhang, Shuhua Zhang, Bomin Yan, Yaoguang Wang, Chunping Xia

**Affiliations:** 1 Surgery, the Affiliated Hospital of Medical College, QingDao University, QingDao.266003. R.P. China; 2 Pathology, the Affiliated Hospital of Medical College, QingDao University, Shan Dong Province, 266003. P.R. China; 3 Molecular Biology, the Affiliated Hospital of Medical College, QingDao University, Shan Dong Province, 266003. P.R. China; 4 Hepatobiliary surgery, Tianjin Medical university Cancer Institute and Hospital, Tianjin, China, 300060, Huanhuxi Road, Hexi District, Tianjin; Key Laboratory of Cancer Prevention and Therapy, Tianjin

## Abstract

**Objective:**

To measure clusterin expression in pancreatic cancer tissues and cell lines and to evaluate whether clusterin confers resistance to gmcitabine in pancreatic cancer cells.

**Methods:**

Immunohistochemistry for clusterin was performed on 50 primary pancreatic cancer tissues and 25 matched backgrounds, and clusterin expression in 5 pancreatic cancer cell lines was quantified by Western blot and PT-PCR. The correlation between clusterin expression level and gmcitabine IC50 in pancreatic cancer cell lines was evaluated. The effect of an antisense oligonucleotide (ASO) against clusterin(OGX-011) on gmcitabine resistance was evaluated by MTT assays. Xenograft model was used to demonstrate tumor growth.

**Results:**

Pancreatic cancer tissues expressed significantly higher levels of clusterin than did normal pancreatic tissues (*P *< 0.01). Clusterin expression levels were correlated with gmcitabine resistance in pancreatic cancer cell lines, and OGX-011 significantly decreased BxPc-3 cells resistance to gmcitabine (*P *< 0.01). *In vivo *systemic administration of AS clusterin and gmcitabine significantly decreased the s.c. BxPC-3 tumor volume compared with mismatch control ODN plus gmcitabine.

**Conclusion:**

Our finding that clusterin expression was significantly higher in pancreatic cancer than in normal pancreatic tissues suggests that clusterin may confer gmcitabine resistance in pancreatic cancer cells.

## Introduction

Pancreatic cancer is resistant to almost all cytotoxic drugs. Currently, gmcitabine appears to be the only clinically active drug but, because of pre-existing or acquired chemoresistance of most of the tumor cells, it failed to significantly improve the outcome of pancreatic carcinoma patients [1].

Clusterin, also known as testosterone-repressed prostate message-2 (TRPM-2), apolipoprotein J (ApoJ), sulfated glycoprotein-2 (SGP-2), and complement lysis inhibitor(CLI), was first isolated from ram rete testes fluid and plays important roles in various pathophysiological processes, such as tissue remodeling, lipid transport, complement regulation, and apoptosis [2]. Initially clusterin has been regarded as a marker for cell death because its expression is up-regulated in various normal and malignant tissues undergoing apoptosis [3-5]. However, recent studies suggest a possible role for this gene in protecting cells from death, and consistently demonstrated that overexpression of clusterin closely correlates with the progression of various human malignancies [6-10]. More recent studies suggest that antisense oligonucleotide or interfering RNAs (siRNAs) to clusterin can enhance chemosensitivity in human cancer cells [11-15]. Taken together, these findings indicate that clusterin may play an important role in chemoresistance. To extend these observations to pancreatic cancer, we measured clusterin expression levels in pancreatic cancer tissue samples and cell lines and sought to determine the role of clusterin in conferring gmcitabine resistance in pancreatic cancer cells.

## Materials and methods

### Cell and Tissue Collection and Preparation

Human pancreatic cancer cell lines(PT45-P1, T3M4, BxPc-3, Capan-1 and PancTu-1) were obtained from the American Type Culture Collection (ATCC, Manassas, Virginia)and maintained in Dulbecco's modified Eagle's medium with 10% fetal calf serum. Pancreas tissue samples [16] (50 tumors and 25 matched backgrounds) were collected and part of the tissues immediately frozen in liquid nitrogen before processing, and part of the tissue was used for immunohistochemical staining.

### Immunohistochemical staining for clusterin

Serial 4-um-thick sections of the tissue array blocks were subjected to immunohistochemical study. The sections were deparaffinized, and endogenous peroxidase was blocked with 3% H2O2. Then the slides were labeled with a monoclonal antibody to clusterin (clone B-5, 1:200 dilution; Santa Cruz Biotechnology, Santa Cruz, CA) for 1 hour. After washing with phosphate-buffered saline, the sections were incubated with biotinylated secondary antibody and then with an avidinbiotin streptavidin-peroxidase complex (Vectastain Elite ABC kit; Vector Laboratories, Burlingame, CA). 3,3'-diaminobenzidine tetrahydrochloride was used as a chromogen, and Mayer's hematoxylin counterstaining was applied. Immunohistochemical staining of clusterin was defined as detectable immunoreaction in cytoplasm. Clusterin expression was scored as follows: negative(-) if no staining was seen or if weak (+) immunoreactivity was observed in <10% of the tumor cells, and positive(overexpression) if >10% of the tumor cells demonstrated moderate (++) to strong (+++) staining. The results of control staining were satisfactory.

### AS Clusterin ODN

The sequences of AS clusterin ODN corresponding to the human clusterin translation initiation site were 5'-CAGCAGCAGAGTCTTCATCAT-3'. A 2-base clusterin MM ODN (5'-CAGCAGCAG AGTATTTA-TCAT-3') was used as a control.

### Treatment of Cells with ODN

Lipofectin, a cationic lipid (Life Technologies, Inc.), was used to increase the ODN uptake of cells. BxPC-3 cells were treated with various concentrations of ODN after a preincubation for 20 min with 3 μg/ml lipofectin in serum-free Opti-MEM (Life Technologies, Inc.). After the beginning of the incubation (4 h), the medium containing ODN and lipofectin was replaced with standard culture medium as described above.

### RNA Extraction and RT-PCR Analysis

The mRNA extraction and RT reaction of the tissue and cell for synthesizing the first-strand cDNA was carried out according to the manufacturer's instructions. The clusterin Primer sequences was:sense:5'-ATGATGAAGACTCTGCTGCT-3', antisense:5'-TCACTCCTCCCGGTGCTT-3', GA PDH: sense: 5'-TGATGGGTGTGAACCACGAG-3', antisense:3'-TTGAAGTCGCAGGAGACA ACC-5'. Fluorescent bands were visualized using a UV-CCD video system (Epi-LightUVFA1100; AISIN COSMOS, Tokyo, Japan) and were analyzed using Quantity One image-analysis software (PDI, NY). The intensity of each band relative to the GAPDH band was represented as the mean ± s.d. The mean ± s.d. values are shown in the figures. P < 0.05 was considered to be statistically significant.

### Western Blot Analysis

Samples containing equal amounts of protein (15 μg) from lysates of the cultured PT45-P1, T3M4, BxPc-3, Capan-1 and PancTu-1 cells and BxPc-3 tumors were electrophoresed on an SDS-polyacrylamide gel and transferred to a nitrocellulose filter. The filters were blocked in PBS containing 5% nonfat milk powder at 4°C overnight and then incubated for 1 h with a 1:400-diluted antihuman clusterin goat polyclonal antibody (Santa Cruz Biotechnology, Inc., Santa Cruz, CA), 1:50 0-diluted antirat β-actin mouse monoclonal antibody (Chemicon International, Inc., Tumecula, CA), The filters were then incubated for 30 min with horseradish peroxidase-conjugated antigoat or mouse IgG antibody (Amersham Life Science, Arlington Heights, IL), and specific proteins were detected using an enhanced chemiluminescence Western blotting analysis system (Amersham Life Science).

### IC50 and MTT assay

Following the addition of 1 × 10^4 ^pancreatic cancer cells into each well of a 96-well plate, 0.1 ml of medium was added, containing various concentrations of gmcitabine. The 50% inhibitory drug concentration (IC50) was obtained by MTT assay. The result of three repeated experiments was presented as the mean ± standard error, and differences were analyzed using the unpaired t.

### Animal Studies

Female C57BL/6 mice at 6-8 weeks old were obtained from Qingdao Medical college, Qingdao University for tumor implantation. All animals were maintained in a sterile environment and cared for within the laboratory animal regulations of the Ministry of Science and Technology of the People's Republic of China (http://www.most.gov.cn/kytj/kytjzcwj/200411). Full details of the study approval by the ethics committee at the affiliated hospital of medical college, Qingdao University. Each experimental group consisted of 10 mice. Each of the tumor cell lines was trypsinized, washed twice with PBS, and injected s.c. with 1 × 10^6 ^cells in the flank as described previously [16]. After injection for 30 days, the diameter in BxPC-3 tumors was 5~8 mm. Mice bearing BxPC-3 tumors was randomly selected for treatment with AS clusterin ODN alone, MM control ODN alone, AS clusterin ODN plus gmcitabine, or MM control ODN plus gmcitabine. After randomization, 10 mg/kg AS clusterin or MM control ODN were injected i.p. once daily into each mouse for 28 days, and 40 *u*M of gmcitabine were injected i.v. twice a week for 2 weeks. Tumor volume was measured once weekly and calculated by the formula: 1/2(length × width × depth). Data points were reported as average tumor volumes ± SD.

### Statistical Assessment

All statistical analyses were performed using SPSS13.0 software. The results were presented as mean ± SD of three replicate assays. Differences between various groups were assessed using ANOVA or Dunnett *t*-test. A P value of <0.05 was considered to indicate statistical significance.

## Results

### Expression of clusterin in pancreatic cancer tissues samples

**Figure 1:**
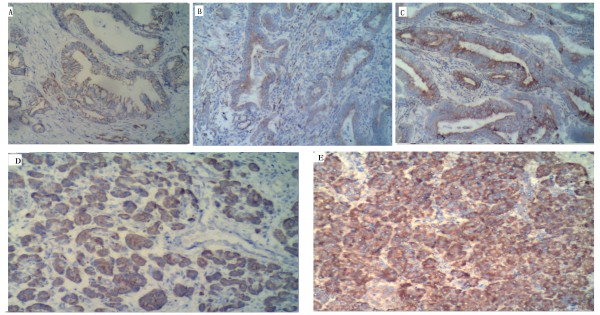
**Expression of clusterin in pancreatic cancer cells**. **A-C,C **lusterin protein expression was detected in the pancreatic cancer cytoplasm. A.(+), B.(++), C.(+++). *D*. clusterin protein expression (+)was detected in the matched backgrounds of the pancreatic cancer. E. Only one patients of the matched backgrounds shown clusterin protein expression (++/+++).

Clusterin protein immunoreactivity was detected both in matched backgrounds and pancreatic cancer cells. The immunostaining results are presented in Table 1. Of the 50 pancreatic cancer tissues, 26 (52%)exhibited clusterin overexpression in cancer cells, 4(8%) exhibited clusterin weak expression in cancer cells(Figure 1.A-C), and no clusterin staining was shown in 20 pancreatic cancertissues. Of the 25 matched backgrounds tissues, Only 1 of these showed strong immunoreactivity (++/+++), and 7 were immunoreactive in 1-10% of the tumour cells (+)(Figure 1.D-E). A highly significant clusterin protein immunoreactivity was shown in pancreatic cancer cells(*P *< 0.05)(Table 1).

**Table 1:** Immunohistochemical staining for clusterin

		Clusterin			
**Groups**	**n**	-	**+**	**++/+++**	***P***
	
pancreatic cancer tissues	50	20	4	26	
matched backgrounds	25	17	7	1	<0.01

The relative clusterin mRNA value was 0.764 ± 0.18 for tumor and 0.14 ± 0.11 for backgrounds. Levels of clusterin was increased in tumor samples in comparison to matched backgrounds tissues (*P *= 0.0136).

### Relationship between clusterin expression and gmcitabine IC50 in pancreatic cancer cell lines

**Figure 2:**
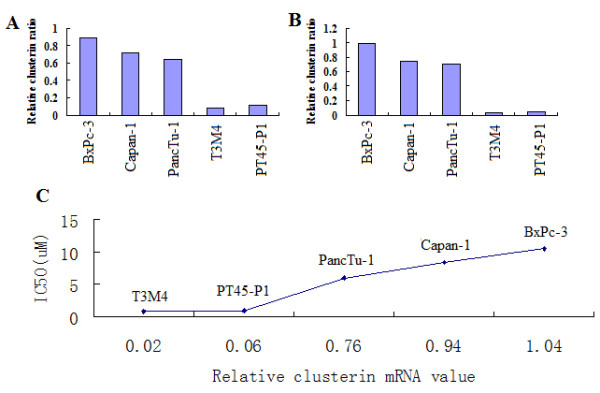
**Expression level of clusterin and gmcitabine IC50 of pancreatic cancer cell lines**. *A*, RT-PCR analysis clusterin mRNA in 5 pancreatic cancer cell lines (PT45-P1, T3M4, BxPc-3, Capan-1 and PancTu-1). *B*, Western blot analysis of 5 pancreatic cancer cell lines (PT45-P1, T3M4, BxPc-3, Capan-1 and PancTu-1) using a monoclonal antibody specific for the clusterin α chain. As a protein loading control, the same blot was incubated with an anti-GAPDH monoclonal antibody. *C*, Correlation between clusterin expression and gmcitabine resistance. Linear regression analysis showed a statistically significant relationship between clusterin expression and gmcitabine IC50 (R^2^, *P *= 0.001).

The association between clusterin protein expression and gmcitabine IC50 was examined in five pancreatic cancer lines:PT45-P1, T3M4, BxPc-3, Capan-1 and PancTu-1. In each of these cell lines, clusterin expression was assayed byWestern blotting and RT-PCR (Figure 2A and 2B). The highest expression of clusterin was observed in BxPc-3 cells. The mean+SD IC50 of gmcitabine for the PT45-P1, T3M4, BxPc-3, Capan-1 and PancTu-1 cell lines was(9.26 ± 0.03) × 10^-7^,(8.38 ± 0.07) × 10^-7^, (1.05 ± 0.09) × 10^-5^, (8.32 ± 0.06) × 10^-6^and (6.04 ± 0.07) × 10^-6 ^M, respectively (Figure 2C). BxPC-3 cells showed the highest resistance to gmcitabine. Thus, gmcitabine protein expression levels showed a significant correlation with resistance to gmcitabine (R^2^, *P *= 0.001).

### AS ODN-mediated Inhibition of Clusterin Expression in BxPc-3 Cells

**Figure 3:**
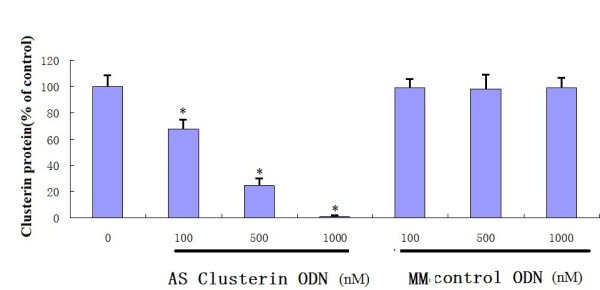
**Sequence-specific and dose-dependent inhibition of clusterin expression by AS clusterin ODN in BxPc-3 cells**. In *A*, BxPc-3 cells were treated daily with various concentrations of AS cluserin ODN or a 2-base clusterin MM ODN as a control for 2 days, total cell protein was extracted from culture cells, and clusterin and GAPDH levels were analyzed by western blotting. In *B*, quantitative analysis of clusterin protein levels after normalization to GAPDH levels in BxPc-3 cells after treatment with various concentrations of AS clusterin ODN or MM control ODN was performed using laser densitometry. Each point represents the mean of triplicate analyses with SD. *, differs from controls (*P *< 0.01) by Student's *t *test.

The effect of treatment with AS clusterin ODN on clusterin protein expression in BxPc-3 cells, which shows the highest level of clusterin expression was evaluated by western blot and RT-PCR analysis. As shown in Figure 3A and 3B, daily treatment of BxPc-3 cells with AS clusterin ODN (100, 500, or 1000 nM) for 2 days reduced clusterin levels by 30, 75, or 98%, respectively. In contrast, clusterin expression was not affected by the 2-base MM control ODN at any of the used concentrations.

### Changes in Clusterin Expression in BxPC-3 Cells after AS Clusterin ODN and Gmcitabine Treatment

**Figure 4:**
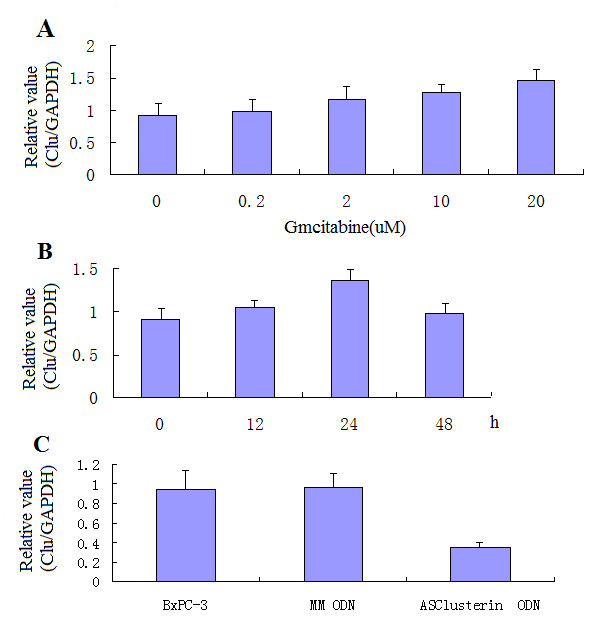
**Effects of AS clusterin ODN and/or gmcitabine treatment on clusterin expression in BxPC-3 cells**. In *A*, cells were treated with various concentrations of gmcitabine for 48 h, total protein was then extracted and analyzed for clusterin and GAPDH levels by western blotting. In *B*, cells were treated with 10 *u*M gmcitabine for indicated intervals, total protein was then extracted, and clusterin and GAPDH levels were analyzed by western blotting. In *C*, cells were treated daily with 500 nM AS clusterin ODN or a 2-base clusterin MM control ODN for48 h. After a 24-h exposure to 10 *u*M gmcitabine, total rotein was then extracted, and clusterin and GAPDH levels were analyzed by western blotting.

Western blot analysis was used to determine the effects of gmcitabine treatment on clusterin protein expression in BxPC-3 cells. As shown in Figure 4A, clusterin induction increased in a dose-dependent manner by gmcitabine treatment at concentrations 0.2-20 *u*M. Time-course experiments demonstrated that gmcitabine-induced clusterin up-regulation peaked by 24 h post-treatment and began decreasing by 48-h post-treatment (Figure 4B). We then examined the effects of combined treatment with AS clusterin ODN and gmcitabine on clusterin expression in BxPC-3 cells. As shown in Figure 4C, 500 nM AS clusterin ODN combined with 10 *u*M gmcitabine for 48 h decreased clusterin protein levels by 60%, compared with 500 nM MM control ODN treatment.

### AS Clusterin ODN Treatment Enhanced Chemosensitivity of BxPc-3 Cells *in Vitro*

**Figure 5:**
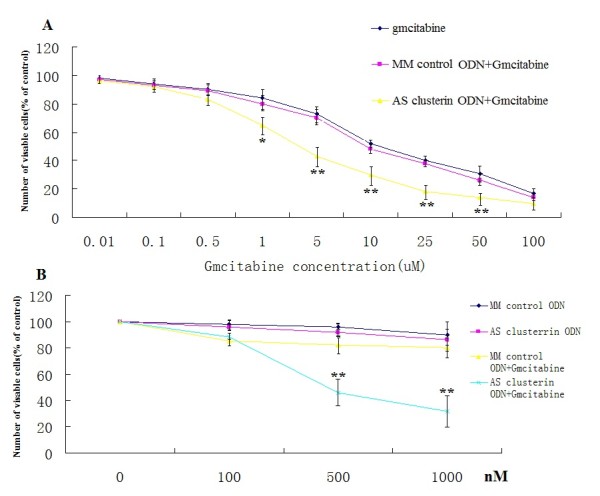
**Effect of combined treatment with AS clusterin ODN and gmcitabine BxPC-3 cell growth**. ***A***, BxPC-3 cells were treated daily with 500 nM AS clusterin ODN or MM control ODN for 2 days. After ODN treatment, the medium was replaced with medium containing various concentrations of gmcitabine. After 48 h of incubation, the number of viable cells was determined by the MTT assay. *B*, BxPC-3 cells were treated daily with 100, 500, 1000 nM AS clusterin ODN or MM control ODN for 2 days. After ODN treatment, the medium was replaced with medium containing 5 uM concentrations of gmcitabine. After 48 h of incubation, the number of viable cells was determined by the MTT assay. Each data point represents the mean of triplicate analyses with SD. ** and *, differs from controls (*P *< 0.01 and *P *< 0.05, respectively) by Student's *t *test.

To determine whether treatment with AS clusterin ODN enhances the cytotoxic effects of gmcitabine, BxPc-3 cells were treated with 500 nM AS clusterin ODN or MM control ODN once daily for 2 days and then incubated with medium containing various concentrations of gmcitabine for 2 days. The MTT assay was then performed to measure the number of viable cells. As shown in Figure 5 A, AS clusterin ODN treatment significantly enhanced chemosensitivity of gmcitabine in a dose-dependent manner, reducing the IC_50 _of gmcitabine by >50%.

To determine whether AS clusterin ODN enhances the cytotoxic effects of gmcitabine was AS ODN dose-dependent, BxPc-3 cells were treated with 100, 500, 1000 nM AS clusterin ODN or MM control ODN once daily for 2 days and then incubated with medium containing 5 uM concentrations of gmcitabine for 2 days. We found AS clusterin ODN treatment significantly enhanced chemosensitivity of gmcitabine in a dose-dependent manner Figure 5B.

### Synergistic Inhibition of Growth of BxPC-3 Cells *in Vivo *by AS Clusterin ODN and Gmcitabine

**Figure 6:**
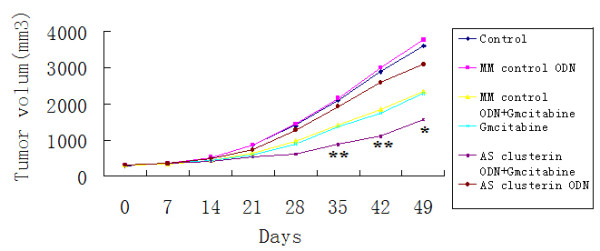
**Effects of combined treatment with AS clusterin ODN plus gmcitabine on BxPC-3 tumor growth**. Mice bearing BxPC-3 tumor were randomly selected for treatment with AS clusterin ODN, MM control ODN, AS clusterin ODN plus gmcitabine, or MM control ODN plus gmcitabine. After tumor cell injection (7 days), 10 mg/kg AS clusterin ODN or MM control ODN was daily injected i.p. for 28 days. gmcitabine (40 μM) was injected i.v. twice a week for 2 weeks. Tumor volume was measured once weekly and calculated by the formula: length × width × depth × 0.5. Each point represents the mean tumor volume in each experimental group containing eight mice with SD. *, differs from controls (*P < 0.01) by Student's *t *test.

The efficacy of a regimen combining AS clusterin ODN and gmcitabine for inhibiting the growth of s.c. BxPC-3 tumors was evaluated. Mice bearing BxPC-3 tumors 5~8 mm in diameter were randomly selected for treatment with AS clusterin ODN alone, MM control ODN alone, AS clusterin ODN plus gmcitabine, or MM control ODN plus gmcitabine. Mean tumor volume was similar at the beginning of treatment in each of these groups. Changes in tumor volume in mice treated with MM control ODN or AS clusterin ODN alone was similar to that of untreated mice. MM control ODN plus gmcitabine was similar to that with gmcitabine alone. It showed AS clusterin ODN alone did not have significant effect on the tumor growth. Although gmcitabine inhibited tumor growth, there was no statistical significance(data not shown). BxPC-3 tumor growth was significantly inhibited by treatment with combined AS clusterin ODN and gmcitabine therapy. After tumor injection (49 days), the tumor volume in mice treated with AS clusterin ODN plus gmcitabine was smaller than that in mice treated with AS clusterin ODN alone, MM control ODN, gmcitabine alone, or MM control ODN plus gmcitabine(,*P < 0.05,**P < 0.01), respectively (Figure 6).

## Discussion

Resistance to anticancer agents is one of the primary impediments to effective cancer therapy. Chemoresistance occurs not only to clinically established therapeutic agents but also to novel targeted therapeutics. Both intrinsic and acquired mechanisms have been implicated in drug resistance but it remains controversial which mechanisms are responsible that lead to failure of therapy in cancer patients [15].

The clusterin (CLU) protein was first discovered more than 25 years ago in rat testis fluid because of its ability to facilitate clustering of a variety of cell types in culture [17]. Since then, homologues of the broadly expressed CLU gene have been identified in several species and CLU proteins have been found in most mammalian body fluids [18]. CLU is an enigmatic molecule, implicated in diverse biological processes, and has additionally been associated with opposing functions in regard to apoptosis. Accumulating evidence from several studies suggests that the pro- and antiapoptotic functions may be related to nuclear and secreted protein isoforms, respectively [19]. The secreted form of CLU is a glycosylated protein of 70-80 kDa that consists of two chains held together by five disulfide bonds, and consequently it appears as a ~40 kDa smear on immunoblots from reducing SDS-PAGE. Its intracellular pre-curser form of 60 kDa may also exhibit an antiapoptotic function [20]. The proapoptotic CLU variant is a 50-55 kDa protein which accumulates in the nucleus of apoptotic cells [19]. How these different CLU protein variants are produced from the CLU gene is poorly understood, although it has been speculated that nuclear CLU results from an alternative splice event skipping exon 2 from the main CLU transcript otherwise translated into secreted CLU [21].

Recent focus has turned to clusterin (CLU) as a key contributor to chemoresistance to anticancer agents. Its role has been documented in prostate cancer for paclitaxel/docetaxel resistance as well as in renal, breast, and lung tumor cells[22-25]. It is noteworthy that only the cytoplasmic/secretory clusterin form (sCLU), and not the nuclear form, is expressed in aggressive late stage tumors, which is in line with its antiapoptotic function [15]. Most significantly, sCLU expression is documented to lead to broad-based resistance to other unrelated chemotherapeutic agents such as doxorubicin, cisplatin, etoposide, and camphothecin [15].

The current treatment of choice for metastatic pancreatic cancer involves single-agent gmcitabine or a combination of gmcitabine with capecitabine or erlotinib (a tyrosine kinase inhibitor). Only 25, 20, 13; 30% of patients respond to this treatment and patients who do respond initially ultimately exhibit disease progression. Median survival for pancreatic cancer patients has reached a plateau due to inherent and acquired resistance to these agents [26]. The actual mechanisms for pancreatic cancer to resist gmcitabine are unclear.

Xie, et al. has found [27] clusterin was overexpressed in pancreatic cancer tissues and pancreatic cancer cell lines, it was not expressed in normal pancreas. We also shown in our study that clusterin expression is significantly higher in pancreatic cancer tissues than in normal pancreatic tissue. To our knowledge, there was no report about whether overexpression enhances their resistance to cytotoxic chemotherapy, and downregulation of clusterin increases their sensitivity cytotoxic chemotherapy. Therefore, in the present study, we evaluated the effect of decrease in clusterin expression in the human pancreatic cancer BxPC-3 cells using AS ODN, and study whether downregulation of clusterin increase their sensitivity cytotoxic chemotherapy both *in vitro *and *in vivo*.

In the present study, we showed a significant correlation between clusterin expression and gmcitabine IC50 in the pancreatic cancer cell lines, we tested the hypothesis that clusterin expression may confer gmcitabine resistance in pancreatic cancer cells. Phosphorothioate AS clusterin ODN corresponding to the human clusterin translation initiation site used in this study inhibited clusterin expression in a dose- and sequence dependent manner, even after gmcitabine treatment, which resulted in an increase in clusterin expression. Furthermore, treatment of BxPC-3(expressing high levels of clusterin) with AS clusterin ODN reduced the IC50 of gmcitabine by > 50%. These findings suggest that clusterin expression in pancreatic cancer cells may confer a phenotype resistant to chemotherapeutic agents;the reduction in clusterin expression by AS clusterin ODN enhances the sensitivity of cytotoxic chemotherapy for pancreatic cancer. Accordingly, based on the findings of the present *in vitro *experiments, we then examined whether AS clusterin ODN therapy synergistically enhances the cytotoxic effect of gmcitabine on the growth BxPC-3 cells *in vivo*. Consistent with the *in vitro *studies, a regimen combining AS clusterin and gmcitabine synergistically inhibited the growth of s.c. BxPC-3 tumors *in vivo*. These findings suggest that it might be possible to achieve powerful cytotoxic effects of gmcitabine at tolerable doses by combining with AS clusterin ODN.

In conclusion, we have shown here that clusterin expression was significantly higher in pancreatic cancer tumor samples than in normal pancreas tissues and that clusterin expression was significantly correlated with gmcitabine resistance in pancreatic cancer cell lines. These findings may lead to the development of new therapeutic regimens, targeting clusterin expression, particularly in patients with gmcitabine-insensitive pancreatic cancers expressing high levels of clusterin.
