# Correction: Clusterin confers gemcitabine resistance in pancreatic cancer

**DOI:** 10.1186/1477-7819-11-149

**Published:** 2013-07-03

**Authors:** Qingfeng Chen, Zhengkun Wang, Kejun Zhang, Xiaoyi Liu, Weihong Cao, Lei Zhang, Shuhua Zhang, Bomin Yan, Yaoguang Wang, Chunping Xia

**Affiliations:** 1 Surgery, the Affiliated Hospital of Medical College, QingDao University, QingDao 266003, R. P. China; 2 Pathology, the Affiliated Hospital of Medical College, QingDao University, QingDao, Shan Dong Province 266003, P. R. China; 3 Molecular Biology, the Affiliated Hospital of Medical College, QingDao University, QingDao, Shan Dong Province 266003, P. R. China; 4 Hepatobiliary surgery, Tianjin Medical University Cancer Institute and Hospital, Huanhuxi Road, Hexi District, Tianjin 300060, China; 5 Key Laboratory of Cancer Prevention and Therapy, Tianjin, China; 6 Department of Surgery, the Affiliated Hospital of Medical College, QingDao University, No 16 Jiangsu Road, Qingdao, Shan Dong Province 266003, P. R. China

## Correction

After the publication of this work [1], we noticed that we had incorrectly used the term ‘OGX-011’. All instances of OGX-011 in the manuscript should be changed to ‘ASO-CLU’.

We apologise to the readers and OncoGenex Technologies for this oversight and any negative effects that may have resulted from it.

## References

[B1] Chen Clusterin confers gmcitabine resistance in pancreatic cancer World J Surg Oncol 2011 11 59 10.1186/1477-7819-9-59 21609464 PMC3120680

